# Securing the Continuity of Medical Competence in Times of Demographic Change: A Proposal

**DOI:** 10.2196/resprot.5897

**Published:** 2016-12-21

**Authors:** Joachim Paul Hasebrook, Jürgen Hinkelmann, Thomas Volkert, Sibyll Rodde, Klaus Hahnenkamp

**Affiliations:** ^1^ zeb.business school Steinbeis University Berlin Muenster Germany; ^2^ University Hosptial Frankfurt Frankfurt Germany; ^3^ Department of Anaesthesiology, Intensive Care and Pain Medicine University Hospital Muenster Muenster Germany; ^4^ zeb.health care zeb.rolfes.schierenbeck.associates Muenster Germany; ^5^ Department of Anaesthesiology, Intensive Care, Emergency Care and Pain Medicine University Medicine Greifswald Greifswald Germany

**Keywords:** shortages of specialist physicians, working life models, flexible working time models, additional medical qualification, promotion of women, combining work and family

## Abstract

**Background:**

University hospitals make up the backbone of medical and economic services of hospitals in Germany: they qualify specialist physicians, ensure medical research, and provide highly specialized maximum medical care, which other hospitals cannot undertake. In addition to this assignment, medical research and academic teaching must be managed despite a growing shortage of specialist physicians. By the year 2020, the need for the replacement of retired physicians and increased demand will total 30,000 positions. The situation will become more difficult because, on the whole, patients are becoming older and sicker and because specialist physicians are able to find more attractive working conditions in smaller hospitals, abroad, or outside of curative medicine.

**Objective:**

In order to retain sufficient qualified employees, major improvements in quality are required in terms of working and training conditions. For this purpose, a sustainable innovation process is necessary, which incorporates solutions from outside of the health care sector in order to be able to learn from experiences and mistakes from other industries. The FacharztPlus project aims to find suitable measures in order to retain specialist physicians for more years after the completion of 5 years of professional training. This should determine the suitability of additional qualifications alongside the professional career and an expertise-related work organization oriented to different stages of life.

**Methods:**

Structured interviews, surveys, and repertory grids are used as preparation for cross-industry expert panels to create future work scenarios for university hospitals. Industries involved are harbor logistics (container terminal), airports, and digitized industrial production (“industry 4.0”) because these industries are also facing a shortage of qualified staff and have to respond to rapidly changing demands. Based on the experts’ scenarios, consensus groups will be established in each university hospital trying to reach consensus about the implementation of relevant factors in order to improve employee retention.

**Results:**

We expect these consensus groups to develop and introduce measures for more structured training procedures, individual and team incentives, organizational guidelines for better recruiting and retention in hospitals, models of flexible and attractive working conditions including shift work and vacation planning, and use of new learning tools (eg, tablet PCs and mobile phones).

**Conclusions:**

All measures are implemented in the Department of Anaesthesiology, Intensive Care, Emergency Care and Pain Medicine at the University Hospital Muenster (UKM) with approximately 150 physicians and in the further 44 departments of the UKM and 22 teaching hospitals, which all together employ more than 5000 physicians. The measures will also be implemented at the university hospitals in Aachen, Rostock, and Greifswald. All decisions and measures will be discussed with representatives from hospital management and professional associations. Results will be presented at conferences and published in journals.

## Introduction

### Hospitals’ Economic Power and Shortage of Medical Staff

Despite the total number of doctors increasing in recent years, a shortage of well-trained doctors is predicted in at least some disciplines for at least 18 countries of the Organisation for Economic Cooperation and Development (OECD) with comprehensive workforce prediction models [1]. The Association of American Medical Colleges predicts, for example, that the United States will need 26% more physicians over the period of 2006 to 2025 [2]. However, the calculated supply of physicians will increase by only 10% to 12% [3]. The European Union sees the issue in the same way: the aging of society and the rising prevalence of diseases related to sedentary lifestyles will increase the burden of disease by 10% to 15% until 2030 (measured as disability-adjusted life years) [3]. The European region is the most affected by noncommunicable diseases and their growth is alarming. Consequently, all European countries need to increase the number of health professionals in the near future. The EU Commission estimates a shortage of 1 million health professionals by 2020, if action is not taken [4]. A lack of health professionals will result in 15% of care services not being delivered due to lack of resources [5].

The shortage of medical staff will continue to advance in coming years. According to details from the German Medical Association, by the year 2019, 18,940 physicians will enter retirement due to their age [6]. These positions need to be replaced, but there are also 5500 that are already vacant and a further 4800 extra positions needed due to increasing life expectancy and patient comorbidity; between 2015 and 2025, the morbidity rate is expected to increase from 5% to 15% [7]. This results in a combined replacement, extra and additional demand for 29,240 medical jobs by 2019. Besides this, the amount of part-time positions will increase by 10,270 during the same period and also add to the open positions. According to more recent studies. Among other reasons, this is caused by the fact that medical doctors are currently predominantly male, while the proportion of females in the workforce will grow significantly in the next few years.

University hospitals have to work under special conditions, because, besides patient care, their employees are also active in research and teaching. There are currently almost no university hospitals that are able to financially combine care, research, and training successfully [8]. In order to be able to comply with the various demands of the future, ensure medical care at a high level, and evade the ever-increasing shortage of medical staff, the staff potentials that are available need to be used more efficiently and sustainably.

### Career Perspectives for Talents

The age structures in all European countries are changing, and together with contracting populations as a whole, the potential labor force will be significantly reduced in the coming decades. The German Institute for Employment Research presents a worst-case scenario in which there is an unsatisfied demand for approximately 6 million open positions in the year 2020 [9]. As stated above, the need for well-trained specialist physicians will continue to rise.

In the regular expert monitor from the German Institute for Vocational Education over 80% of the surveyed personnel managers report a considerable worsening of education levels due to errors in spelling and abilities to cope with a workload [10]. More and more industries are noticing the change from an “employer’s market” to an “applicant’s market,” on which applicants rather than employers influence the market [11]. Bernhard Marschall, academic dean of the faculty of medicine at the University of Muenster, wrote in a major German newspaper (Frankfurter Allgemeinen Zeitung, F.A.Z.) on September 22, 2011: “While medicine used to be seen as an attractive career with good opportunities to earn well, now the focus is on job security, compatibility of work and family and the high social image.” In rankings of school and university leavers, it is easy to see a further focus on fewer courses of study [12,13]. Finally, there are many other alternatives to curative work in German hospitals that compete for well-trained medical students. Curative work is often abandoned due to more attractive working conditions elsewhere, in particular in health care management, external assessments, commercial research, but also for caring for their own child [14]. Of the approximately 17,000 medical staff who were trained in Germany, working abroad caused a training investment of approximately €350,000 per graduate to be lost [15,16].

In consequence, a new focus must be on the value and performance culture and an ongoing exchange of interests of economic and performance-based demands of hospitals with the individual demands of staff [17]. This will lead to a “democratization of talent management” [18] bringing individualized job descriptions and flexible competence management to the top agenda of human resource management [19,20].

### Work-Related Values: A Generational Comparison

This “democratization of talent management” can be traced to the changed values of today’s working generations [21]: (1) baby boomers (born 1955-1969), who are reliable at dealing with competition and conflict due to coping with high birth rates, high awareness of loyalty and duty (live to work); (2) Generation X (born 1970-1980), resistant to stress, motivated, pragmatic, and willing to train further, the combination of private life, family, and work is especially important (work to live); and (3) Generation Y (born 1981-1995), expects a performance orientation and a “feel-good culture,” which is said to promise a consumption-oriented attitude to work and limited loyalty with a great need to achieve self-fulfillment (live, even at work).

This assessment of different generations has been detailed by a study by German studies [22] and has been confirmed by international research [23]: “youth materialism” (ie, possessing money and status objects) grew over the time and reached its peak in Generation X at the beginning of the 1990s and has remained persistently high until the generation born after the turn of the century (so-called Generation Me or Z). While these materialistic values increased, the predominant value of work (so-called work centrality) continued to decrease, which indicates a growing gulf between material demands and willingness to work for them [24]. Today, hospitals are not just faced with an applicant’s market, but also a new understanding of work and new demands for leadership and participation [25]. These are characterized by a greater desire for communication, teamwork, and appreciative leadership style and a high level of pragmatism [26].

The fact that the work environment and especially communication between leaders and staff is changing fundamentally is only slowly being accepted by the medical and financial management levels in hospitals [27]. Leading doctors and managers in hospitals have noticed that dealing with young doctors and job starters is becoming much more time-consuming and complicated, and that established structures and the organization of work is being questioned. The necessity to work overtime, the quality of training, and the style of communication have all come under fire: the change from an employer’s market to an applicant’s market demands far-reaching changes in personnel management, which most hospitals are currently not yet ready for.

### Need for Intergenerational Work in Hospitals

While baby boomers often occupy the managing positions in hospitals and members of Generation X can look back on an established career and possibly their own family life, Generation Y represents the emerging talent for highly qualified specialists and managers in hospitals [28]. This generation has high demands for the material equipment and flexibility of the work. Another aspiration is the higher importance of family and the growing wish of young men to relinquish their traditional role of “breadwinner” and to experience their fatherhood [29,30]. However, it is not men, but rather women, who represent the future majority of medical staff. The share of female medical students has continued to grow over the years and, according to recent German education report, is approximately two-thirds, the future of the medical profession is thus clearly female [31]. The working environment is currently still very far removed from the ideals of young women [32] and family life is also only moving slowly toward an equal time division and workload for men and women [33].

The measures that have been taken so far are neither enough, nor sufficiently long-term, to be able to counter the problems on the labor market resulting from demographic change, requirements of new generations, and the increasing share of female medical staff. The FacharztPlus project, therefore, shall create improvements in the following areas for flexible work, better life-long working perspectives, and continuous improvement to specialist work in hospitals:

1. The abandonment of patient care must be slowed down and reduced: this exit from curative work is often due to more attractive working conditions especially in health care management, external assessments, commercial research, and child care [14].

2. The appeal of medical employment in Germany needs to be raised in comparison to foreign employers in order to reduce physician migration abroad, and thus to reduce the loss of training investments (especially important in regions near national borders such as Muensterland): 17,000 medical staff who were trained in Germany work abroad, by the year 2019, this will have increased by 11,300; so that the training investment of approximately €350,000 per graduate is lost [34].

3. Experienced staff members and the expertise that they hold must be used more effectively for experiential learning on-the-job and optimal patient care. For example, this can mean that experienced specialists with particular knowledge can share it better in special clinical cases.

4. Medical care and career qualification must offer close interaction and longer-term perspectives in order to transform the idea of “forwarding” specialists from university hospitals to regional hospitals toward a principle of “additional qualification” at university hospitals after completion of specialist examinations.

5. The combination of work and family must be improved, especially for female specialists, by offering more flexible and individually adjustable working models (eg, in part-time work) [15].

### Objectives

#### Life-Long Working Perspectives as an Aim

The FacharztPlus project aims to develop and test measures in order to gain specialist physicians for 5 or more years of further work after completion of their 5 to 6 years of professional training. The project aims to ensure that especially female specialists find work and career perspectives at university hospitals or other hospitals upon completion of their 5-year specialist training for at least the same time again. The core of the project is expertise-based staffing in order to join medical care and further qualifications together. While such models have already been successfully implemented in other industries, such as production management, there is currently no link between generally short-term expertise-based staffing and long-term, flexible work and career planning—neither in hospital operations, nor in any other industry. Exactly this combination makes up the specialist core of the FacharztPlus project and will be systematically investigated and tested for hospitals first. Beyond this, the concept will then be transferred to other industries.

#### Flexible and Individual Work for Life-Long Working Perspectives

Shift work with high pressure and often night duty, as well as changing demands at short notice displace especially older and more experienced specialist physicians from the extremely experience-dependent world of university medicine. So flexible duty rosters that can be changed at short notice are being developed to ease medical staff. For this purpose, large methodological and technical obstacles would need to be overcome in long- and short-term staffing (eg, through simulating personnel scenarios and electronic shift exchange portals) and additional services would need to be provided in order to ease the workload of specialist physicians (eg, for stress reduction). There is currently no personnel software on the market, which is tailored to the specific needs of staffing in university hospitals, and accordingly, there is no related personnel and organizational guide. The necessary information technology (IT) and technical concepts for this purpose are being developed and tested as part of the project.

#### Life-Long Qualification for More Attractive Careers

The university hospital in Muenster sets standards for student training, for instance with its own student hospital and the unique, live three-dimensional–simulated learning environment, which allows simulated rescue work in a German university hospital, for example. However, the expectations that this raises are not fulfilled in the subsequent training to become a specialist. There simply is no subsequent “specialist physician curriculum.” The additional qualification should be made more appealing through attractive forms of self-managed learning and practical knowledge management (eg, through social software and e-learning portfolios). By involving academic experts as well as networks, the additional qualification is expanded to a sustainable qualification platform and—where feasible and meaningful—consolidated into a curriculum.

#### Expertise-Based Staffing to Combine Medical Care and Further Qualification

Staffing mostly occurs based on availability and presence. Certain special competencies, such as anesthetics for infants, will not follow this staffing method, as they require special training. The opportunity to learn while working in special medical cases is not used enough. Thus, the aim is to establish expertise-based staffing, based on recording competence groups (eg, application fields, process groups) and competence levels (eg, “only under supervision,” “independent,” “can supervise others”) and oriented toward comparable models, such as those from production management. This kind of staffing allows optimal utilization of available expertise, but also more targeted planning of additional qualifications, for example, specialist physicians that can supervise others might always work with colleagues that need to gather experience in a certain situation.

#### Ongoing Optimization and Innovation Processes

It is difficult to initiate, implement, and sustainably establish changes. This is made more difficult in university hospitals due to fluctuations of specialists and training rotations. The FacharztPlus project aims to establish a process model for sustainable innovation processes at University of Muenster (UKM) that is based on interdisciplinary exchange and establishing fixed-communication structures in internal and external networks. Through flexible organization of work, personal flexibility should also be established so that as many physicians as possible can take part in innovation processes.

#### Specialist Consulting

A range of specialist consulting services that currently does not exist on the market is being developed; these will include flexible learning and working conditions in hospitals with related implementation support regarding change management and personnel and IT concepts. The specific demographic developments for medical care make a consistent legal, organizational, and IT solution for hospitals necessary, but this kind of solution does not currently exist. However, the solution does not just need to be feasible in terms of personnel, technology, and organization, but it must also be able to be financed through billable compensation. In that respect, it must be considered that the continued medical training, as provided at university hospitals, is not financed through lump compensations (German diagnosis-related groups). The project will thus involve developing a financially feasible package of measures based on IT and technical concepts. These concepts must allow innovative, IT-supported learning scenarios and their evaluation. For this purpose, a form of education management and controlling that is somewhat new for hospitals must be developed.

## Methods

### Learning From Other Industries

The implementation strategy and process in the FacharztPlus project are based on the basic presumption that a hospital uses solutions for flexible learning and working conditions only to a limited extent. Through a cross-sector search, more solution approaches come into view, but they have not been trialed in daily hospital work, and thus need to be exactly tested, adjusted, and checked for their day-to-day feasibility. This basic consideration results in a stepwise comparison and improvement process (Textbox 1).

**Stepwise cross-industry comparison and improvement process.**

1. Comparison of working and career models between industries:

Solutions that are used in hospitals are professionally, methodically, and technically insufficient for effectively countering the shortage of specialist physicians. Framework conditions, planning, and implementation and successes of new working and career models are thus compared in an industry panel and selected for further assessment and testing.

2. Cross-sector transfer of strengths and selection of measures:

Measures that may be worth considering for university hospitals are identified in a meta-analysis based on quality standards. Quality standards that are relevant in hospitals form the basis (cooperation for transparency and quality in health care [35]). The result consists of measures that are checked for their quality from the point of view of a hospital.

3. Review of measures for labor law, organization, and technology:

The selected measures are subjected to a thorough review of labor law and organizational and technical factors. For this purpose, various implementation scenarios are developed and reviewed by the institute for innovative working conditions in hospitals in terms of their feasibility pertaining to labor law. An appropriately staffed academic project council that also supports the planning and evaluation of measures carries out an academic review. Subsequently, UKM carries out a personnel, organizational, and technical review. The industry panel, quality assessment, and review criteria and results are documented in an interim report.

4. Planning, implementing, and evaluating measures:

UKM supports the development of an implementation and evaluation plan that will be implemented in a pilot trial in the hospital. Every 2 months, a progress and success assessment will be made regarding personnel, technical, and financial factors. Depending on the implementation progress and success, during the pilot trial, measures can be aborted, changed, or added in order to achieve implementation successes. If initial implementation successes are achieved at UKM, then further departments will be added. This applies to the other departments at UKM, but also to implementation partners that have also been added to the project.

5. Use and development of internal and external networks:

In order to gain other hospitals for the implementation of measures, the pilot trials and their evaluation results will be presented to the UKM departments, centers, and institutions as well as teaching hospitals as part of regular UKM events. Hospital and professional associations, specialist publications, and medical congresses that take place during the project schedule will be used to spread the results and experiences. The specialist program council plays a major role both in the assessment and the spreading of project results, especially of implementation successes in the hospitals. This council is made up of representatives from the leading associations from the health care sector and medical self-government.

### Accompanying Research

The accompanying research is aligned with the implementation strategy. First, it is of preeminent importance to better understand personal reasons for retention and change. Simple surveys do not help to uncover hidden motives or give an insight in the long-term development of personal reasoning. Therefore, we apply participatory assessment methods and personal construct theory to employees along the “life cycle” in a hospital: from assistant physician in early training stages to experienced supervising physicians and persons, who left the hospital. Second, we integrate a great number of experts from industries with similar challenges, that is, companies employing highly qualified staff and dealing with quickly shifting working demands. We are applying scenario technique and focus on experts from airport management, harbor management, and “Industry 4.0” (Germany’s digital industrial production) in order to achieve new insights and ideas to improve the work organization in hospitals. Third, we create a test bed for the measures inspired and created by the experts from other industries in order to ensure transferability of all measures. The test bed has three stages: (1) experimental environment in which the main project partner, University Hospital Muenster, tries out early versions of the measures (eg, a new guideline to employee interviews); (2) reality check of the measures that will introduced in some parts of the hospital (eg, new employee interviews for one specific group of physicians); and (3) roll-out to the entire hospitals as well as transfer to other hospitals, namely the project partners and University Hospitals in Aachen, Rostock, and Greifswald. The transfer is a test in itself, what measures and to what extend measures can be transferred from hospital to another.

#### Retention Factors and Personal Constructs

**Figure 1:**
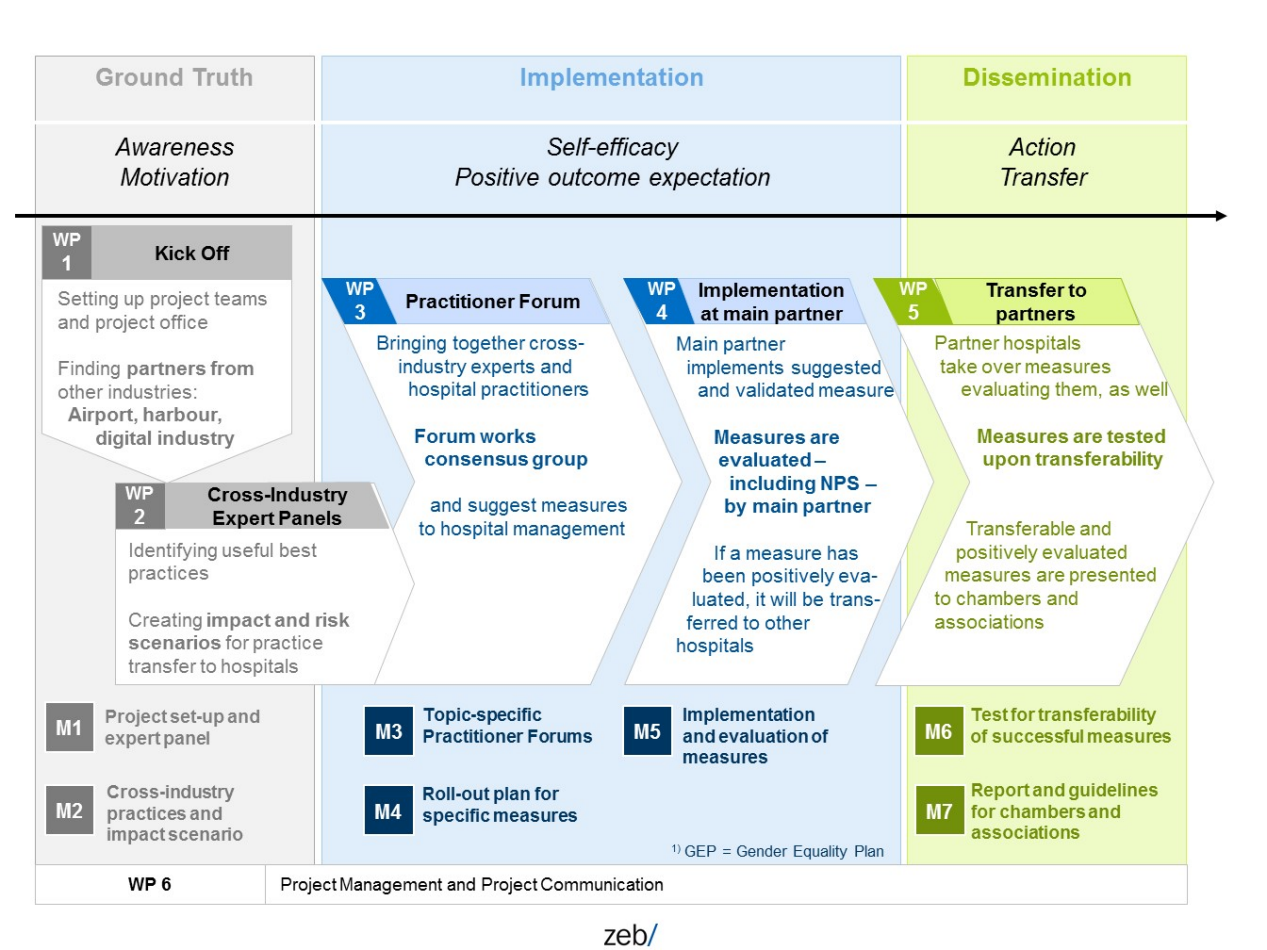
Project plan and milestones of FacharztPlus project.

**Figure 2:**
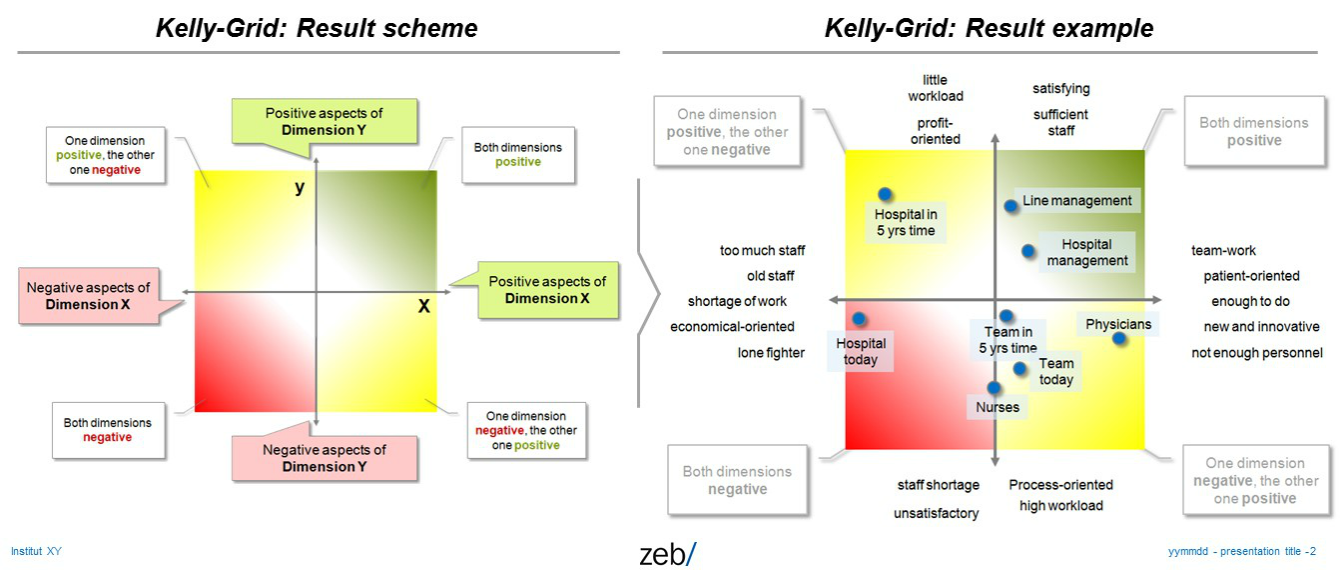
Visualization scheme for Kelly Grids (left) with sample result taken from FacharztPlus project (right).

We want to apply methods that combine qualitative and quantitative aspects as well as personal views and institutional variables. In a first step, we interview a sample of approximately 30 physicians in order to extract major personal aspects of retention and change motives. In a second step, we derive a structured Web-based survey based on these factors and apply it to all more than 300 physicians employed at the 4 university hospitals involved in the project. In a third step, we try to understand “personal constructs” by using Kelly Grids (or repertory grids) to visualize personal construct spaces (Figure 1) from different target groups, including young professionals in their training phase, experienced physicians, medical management, and persons who left the hospital and work elsewhere. Repertory Grids is a computer-based survey that combines qualitative and quantitative elements: interviewees respond to predefined elements, such as “nurses,” “physicians,” and “hospital management,” and decide whether these elements are “similar” or “different.” If they are different, interviewees give a short explanation, why they are different (eg, physicians are “flexible in planning and decision making”) and the opposite (eg, “bureaucratic, inflexible decision making”). In a second step, all predefined elements are rated from 1 to 10 on all scales, which have been created in the first step (eg, on the scale from 1=flexible to 10=bureaucratic, physicians get a 3 and hospital managements is rated 9). This technique has been successfully applied to various aspects of work life in hospitals [36]. It allows for mapping out of “mental landscapes” showing the mental distance between elements, with what terms they are described, and whether they are rated more positively or negatively [37] (Figure 2).

#### Expert Panels

**Figure 3:**
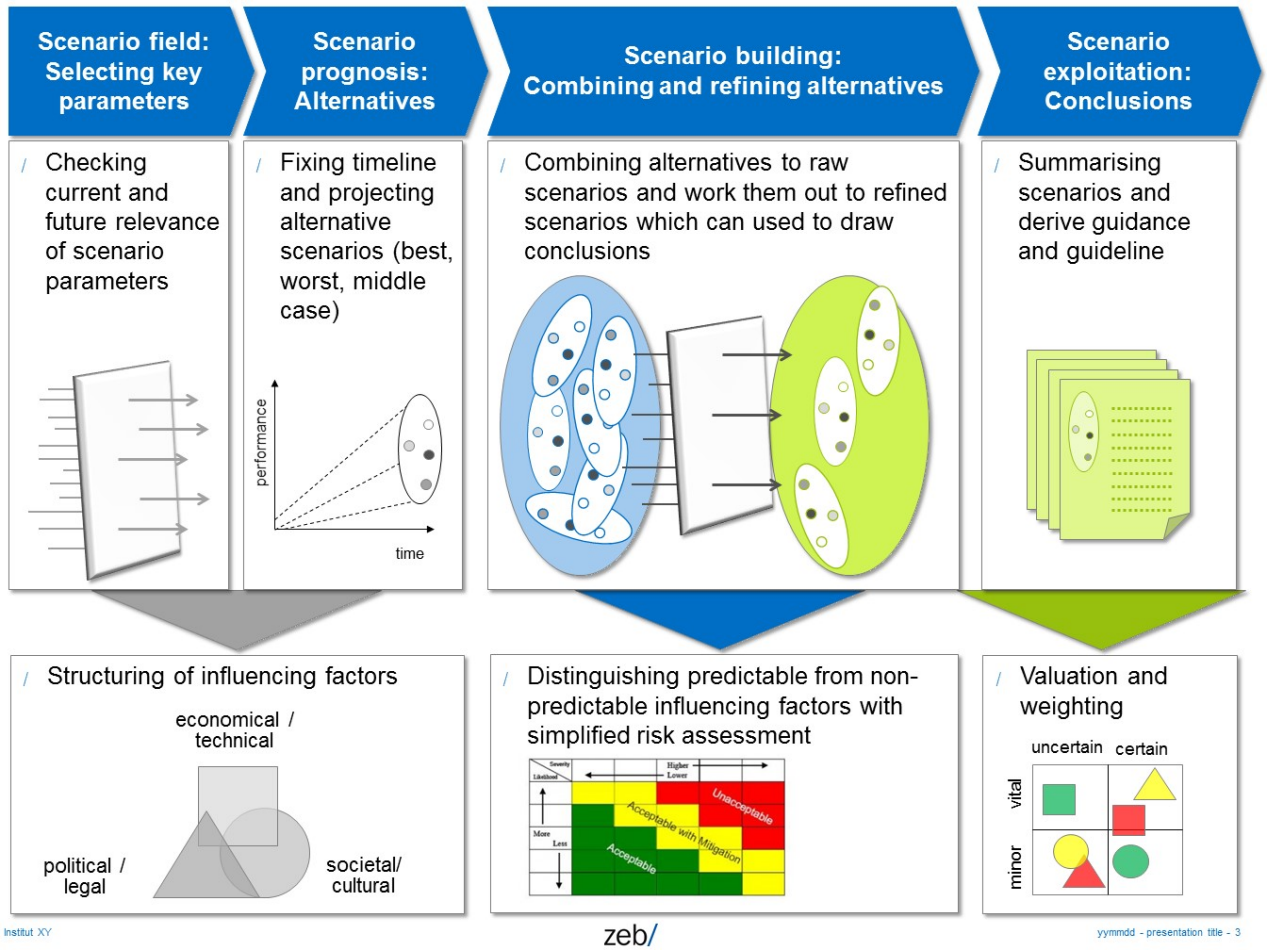
Full (top) and abbreviated (bottom) form of scenario analysis for workshops and expert panels. (Source: FacharztPlus project.)

The expert panels should bring together human resource (HR) managers from airport management, harbor management, and “Industry 4.0.” We start with onsite visits and separated expert panels helping us to understand how HR is handled in the different industries and what practice may be transferred to work life in hospitals. After having identified experts and useful practices, we shall invite experts from these industries and practitioners from hospitals to attend “practice forums.” These forums will apply an abbreviated form of a “scenario technique,” which is frequently applied to strategy and restructuring processes [38]. More specifically, scenario technique is used for impact and risk analysis in health care processes [39]. We will apply an abbreviated form of scenario analysis, which has been developed and used by the German Ministry of Education and Research, for instance, for technical options assessment [40] (Figure 3). As a result a these workshops, we expect stimuli and best practices from other industries as well as impact and risk scenarios concerning the transfer of these practices from other industries into hospitals.

#### Planning, Testing, and Dissemination

Based on the scenarios, the main research partner, University Hospital Muenster, establishes different workgroups consisting of 6 to 8 participants and taking 3 to 5 meetings to come up with full consensus. The measures suggested by the groups will be presented to and confirmed by the university hospital’s management. We apply the Nominal Group Technique (NGT) in order reach and document consensus about necessary organizational transformational measures. Research has shown that NGT is superior to moderated discussions in focus groups and other more structured techniques, because it regards individual differences, accounts for the strength of conviction, and documents the progress toward achievement of consensus [41]. Moreover, NGT is well-documented [42], easy to learn and apply [43], as well as largely applied in health care and nursing [44]. Nominal grouping is a highly structured technique designed to maximize the individual contribution of each respondent. Comparative studies reveal that nominal group members produced a significantly larger amount of enhancements than respondents in focus groups and show greater levels of group member satisfaction [41]. The different steps of the NGT documents individual inputs, as a list of ideas, and group decisions in the form of ranking tables. Therefore, ideas and decisions can be easily validated, shared, and used as input for further refinement.

**Figure 4:**
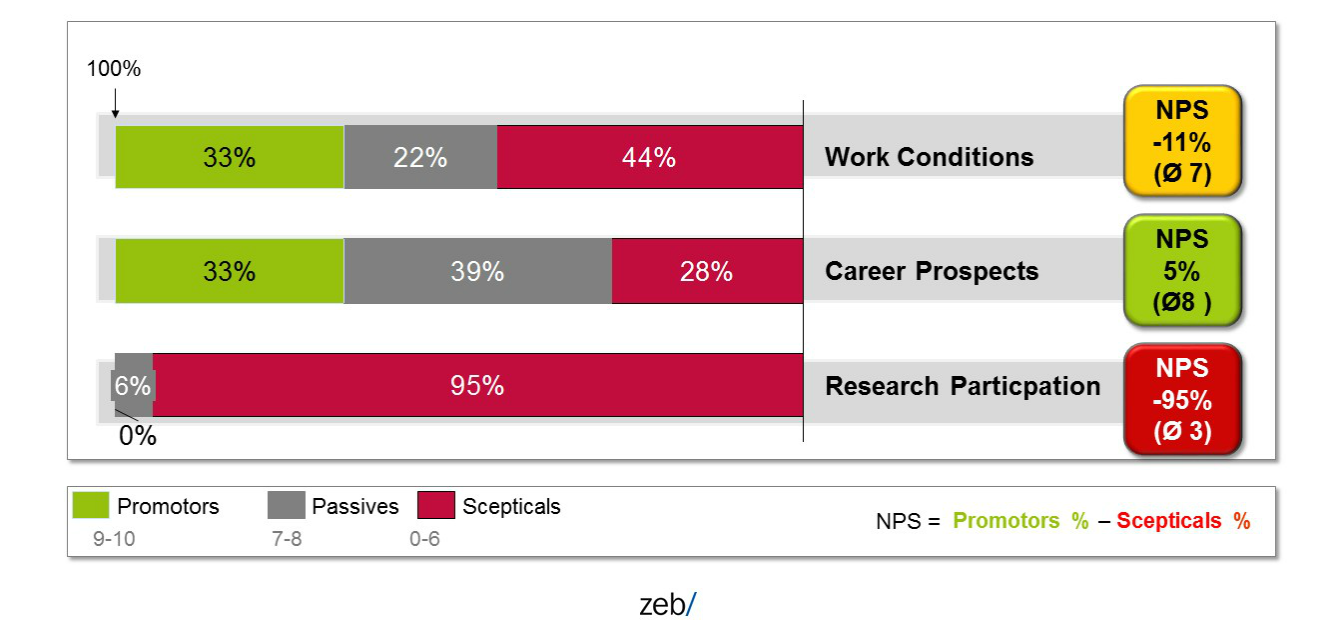
Net Promoter Score showing the percentage of physicians in favor of or against working, career, and research conditions in a university hospital.

As soon as a measure has been implemented to the main research partner more consensus groups are initiated at the partner university hospitals in Greifswald, Rostock, and in Aachen. As soon as a new measure is being implemented, the percentage of persons promoting the new measure as compared with the percentage of skeptical people is constantly measured via the Internet. This proportion is derived from customer loyalty metrics called “Net Promoter Score” [45] and gives a clear indication to what extend gender equality is promoted within the hospital (Figure 4).

## Results

### Preparing and Conducting the Project

During the reviewing process of the research proposal, the reviewers made several suggestions for improvements. For better academic support and spread of project results, the reviewers recommend establishing a specialist and academic council. In addition, the social partners are to be involved into the project. Therefore, an advisory council has been established and works alongside the steering committee, which itself is made up of managers from the UKM and Department and a partner from zeb. The advisory council consists of representatives from medical self-government (medical associations), professional associations (German society for anesthesiology and intensive care), the project sponsor (central federal association of health insurance funds), and hospital associations (German Hospital Federation, DKG, and special purpose associations). The council meets at least twice per year and is informed about project progress on a quarterly basis. In addition, the project management, consisting of 1 representative from UKM and 1 from zeb, is supported by an academic supervisory council, which methodically provides consultation and reviews in topics, such as work organization and development of competence and organization.

The possibility to transfer to other university hospitals is to be ensured through the selection of suitable implementation partners and potentially including further hospitals as implementation partners. Therefore, university hospitals in Greifswald, Rostock, and Aachen were gained as implementation partners. The hospitals offer all modern procedures of general and local anesthesiology and possess proven expertise in the area of anesthesiology and intensive therapy support for high-risk patients. Each year, these implementation partners conduct more than 50,000 cases of anesthetics. It remains possible that further implementation partners (eg, regional hospitals for basic medical care) be added during the course of the project.

### Expected Project Results

The high pressure to act due to the growing shortage of specialist physicians, the beacon function of the UKM, and the close involvement of many medium-sized and regional hospitals and of associations in the course of the project, as well as the information offered by it encourages other hospitals to test flexible working and career models. This will prove and incrementally improve the transferability of the procedures and measures of FacharztPlus project, which are built on the following material project results: (1) industry panel–airport, sea harbor, automotive, financial services, and health care sectors–to measures for flexible working and career models for highly-qualified executives with additional need for qualification; (2) quality review of the industry panel in terms of planning, implementation, review, and sustainability; (3) comprehensive review of hospital-relevant measures with regard to organizational, technical, and labor law-related aspects; (4) implementation consulting and support for hospitals that want to introduce flexible and more sustainable working and career models; and (5) reliable data on acceptance, performance capacity, recommendation levels, and intentions to stay (especially for female specialist physicians).

### Exploitation of Project Results

#### Commercial Exploitation

Economically, the optimal utilization of available specialist physicians allows for optimal utilization of surgery capacities and greater patient security. A higher retention of specialist physicians through more opportunities for additional qualification and more flexible working hours reduces the costs for familiarization and unwanted fluctuation. A greater attractiveness of work reduces the costs associated with searching and recruiting. A further cost reduction is achieved through higher process efficiency and consistent data in human resources. FacharztPlus develops an organizational guide for hospitals and a criteria catalog for selecting and using personnel software at hospitals. A consulting portfolio for sustainable improvement of medical working conditions will be created together with the professional associations and medical councils.

#### Economic Benefits for University Hospitals

University hospitals can only offer specialist physicians an attractive life-long working perspective if flexible working conditions (even in part-time work) are associated with additional qualifications that are linked to specific experiences (eg, workforce planning and placement, which allows to work with rare clinical cases). Such a close combination of working conditions, personnel resources, and competence development is decisive for recruiting and retaining, but also for cost-carrying utilization of highly-specialist and highly qualified medical staff. Approaches for solving this core problem in hospitals, thus, have several cost effects at the same time (Textbox 2).

#### Academic and Technical Exploitation

When demographic factors are considered, the academic interests in concepts and measures for better recruiting, retaining, and training of specialist physicians are enormous. Already through the discussion of the FacharztPlus project, invitations arose to prepare specialist medical papers and to attend scientific conferences. Through utilization of the project, substantial contributions can be expected on several issues (Textbox 3).

**Expected cost effects of improved employee retention.**

- The costs for recruiting fall because the employer appeal increases through the publications on the project and their long-term effects even after project completion. Currently, the recruiting costs amount to €20,000 to €80,000 per position. With total recruiting budgets of €200,000 to €500,000 per department, even low-percentage savings are very valuable.
- Increased retention reduces the demand for new and replacement staff. This means that the recruiting costs will not just fall, but more and better training can be conducted with higher numbers of cases and better quality treatment. This supports the UKM and the department on the profit side and increases the appeal in the further training and additional qualification of specialist physicians.
- Through optimal staffing, not only will the treatment and training quality increase, but also the utilization of the most expensive rooms of the department—the operating rooms. Previous experience in operating room management at other hospitals shows considerably reduced set-up times before the first incision, improved time from skin incision to closure (ie, duration of operations), fewer complications, and higher satisfaction of medical personnel due to a better capacity to plan staffing.
- This improved staff planning should also lead to remuneration that is fair considering performance and qualification; although this is theoretically possible through tariffs, it is currently uncommon in practice. This means, for example, that in large proportions of on-call work, more than 49% of the time is worked, meaning that this should be paid at full rate and not at on-call rates. Better planning and clearer remuneration standards should increase the profitability and employer appeal at the same time.

**Expected organizational adaptations for improved employee retention.**

- Structured training, even on-the-job, with appropriate, planned flexibility based on the specific staffing experiences of UKM and the value partners will be published in professional magazines.
- Questions regarding hospital financing including incentives for the hospital operators to provide resources for further training will be discussed with the associations and recommendations will be prepared in practice forums for hospital and special purpose associations.
- Specific measures will be recommended in the organizational guide for helping improve recruiting and retention in hospitals. Implementation experiences and successes will be published in specialist articles.
- Models of flexible and attractive training will be published in specialist articles and presented to an expert audience on professional conferences.
- Long-term change and adjustment requirements are being discussed with representatives from hospitals and professional associations in practice forums and recommendations are being derived. Implementation experiences will be published in specialist articles.
- To make allowances for the learning behavior of new generations, the use of new learning tools (eg, tablet PCs and mobile phones) and new training forms new forms of cooperative and media-supported learning will be developed.
- To achieve a structured and demand-oriented training, better personnel planning software, and electronic skills management systems are needed: in a standardized process, suitable software providers will be identified and with 1 of these software providers, a software solution will be implemented at UKM. IT solutions, once they have been developed, will be available to all hospitals.

## Discussion

### Project Status

Overall, utilization of the project results will lead to short-, middle-, and long-term effects. In the short-term, that is, during the project execution, an organizational guide with sample calculations for investment needs and cost reductions through flexible work and training offers is being developed, a series of events called “demography and competence management in hospitals” has been initiated together with the associations and interest groups represented in the project council [46] several articles have been published in practice-oriented journals [47] and volumes [48]. In addition, a personnel concept and an IT concept for a software program for flexible personnel planning in hospitals are under development. In the mid-term, within 2 to 3 years of project completion, we aim to develop recommendations for supporting flexible working and learning conditions in hospitals through associations based on project results documented in an organizational guide, to initiate a national platform for “demography and competence management in hospitals,” based on the event series. Moreover, we prepare the publication and marketing of the new consulting portfolio for hospitals. In the long-term, we aim to develop recommendations for adjusting and opening tariff contracts and for financing flexible work and training offers in hospitals as part of the statutory payment system. Innovative software for personnel planning in hospitals will be implemented and tested as a new software package through software providers.

### Outlook

In association with interest groups and medical associations FacharztPlus will support the structuring of further medical training through better controlling of training, and standardization of “learning analytics.” The research project also promotes competence-based professional training instead of collecting “training credits” and helps to adjust education and further training of the medical profession to reflect current challenges in curative medical care (eg, increasing comorbidity). A special focus lies on the incorporation of gender-specific features into the job profile in order to be able to use more work hours from qualified and experienced female specialist physicians through the development of new gender- and generation-specific working time models. Finally, we want to motivate German health care policy to strive for tariff policy and regulatory framework conditions, which encourage flexible work and training as well as models of future financing of further specialist physician training and subsequent training for additional qualification of specialist physicians.

## Abbreviations

IT: information technology
HR: human resources
NGT: nominal group technique
UKM: University Hospital of Muenster
